# Not enough there, too many here: understanding geographical imbalances in the distribution of the health workforce

**DOI:** 10.1186/1478-4491-4-12

**Published:** 2006-05-27

**Authors:** Gilles Dussault, Maria Cristina Franceschini

**Affiliations:** 1 Human Development Department, World Bank Institute, Washington, DC, USA; 2 Consultant, Pan American Health Organization, Washington, DC, USA

## Abstract

Access to good-quality health services is crucial for the improvement of many health outcomes, such as those targeted by the Millennium Development Goals (MDGs) adopted by the international community in 2000. The health-related MDGs cannot be achieved if vulnerable populations do not have access to skilled personnel and to other necessary inputs. This paper focuses on the geographical dimension of access and on one of its critical determinants: the availability of qualified personnel. The objective of this paper is to offer a better understanding of the determinants of geographical imbalances in the distribution of health personnel, and to identify and assess the strategies developed to correct them. It reviews the recent literature on determinants, barriers and the effects of strategies that attempted to correct geographical imbalances, with a focus on empirical studies from developing and developed countries. An analysis of determinants of success and failures of strategies implemented, and a summary of lessons learnt, is included.

## Introduction

Access to good-quality health services is crucial for the improvement of health outcomes, such as those targeted by the Millennium Development Goals (MDGs) adopted by the international community in 2000. For example, the reduction of maternal mortality by 75% in 2015 depends on access to skilled care at birth and during the pregnancy [1,2]. But often, services are not available at a reasonable distance; or they are available, but people cannot afford them. Or, they are not accessible for some organizational reason, such as limited hours of presence of staff, unfriendly behaviour towards users, cultural barriers, and so on. Accessibility of health services is a multidimensional concept [3] that refers to geographical, economic (affordability), organizational and cultural (acceptability) factors that can facilitate or hinder use of services.

This article will focus on the geographical dimension of access and on one of its critical determinants: the availability of qualified personnel. There are many examples of poor countries that provide good coverage of their territory with health facilities yet limited access to services, because facilities lack the personnel needed to function normally. A well-balanced distribution of infrastructures needs to go hand-in-hand with a well-balanced distribution of health personnel to be worth the investment, let alone to have an impact on the health of the population.

### Geographical imbalances: a widespread problem

Unbalanced distribution of health personnel between and within countries is a worldwide, longstanding and serious problem. All countries, rich and poor, report a higher proportion of health personnel in urban and wealthier areas. In Nicaragua, around 50% of the health personnel are concentrated in the capital, Managua, which comprises only one-fifth of the country's population [4]. In Mexico, it is estimated that 15% of all physicians are unemployed, underemployed or inactive. Yet despite this apparent surplus, rural posts remain unfilled [5]. Indonesia's vast size and difficult terrain presents an enormous obstacle for the delivery of health services and for a balanced distribution of health personnel. Doctors and nurses are reluctant to relocate to remote islands and forest locations that offer poor communications with the rest of the country and few amenities for health professionals and their families [6].

In Bangladesh the metropolitan areas contain around 15% of the country's population but 35% of doctors and 30% of nurses, in government positions. Since there are virtually no doctors or nurses in the private sector outside the metropolitan areas, the geographical concentration of these providers in the metropolitan areas is even greater [7]. In Brazil in 1995, the number of physicians per 1000 population by region varied from 0.52 and 0.66 in the poorer regions of the north and the northeast to 1.75 and 2.05 in the states of São Paulo and Rio de Janeiro, in the richer southeast region. The average for the whole country was 1.19. This gap in favor of richer regions is smaller than it was 25 years earlier, thanks to efforts to expand the coverage of the population by public services. But "the low incomes of the population have discouraged the settlement of doctors" in the poorer regions [8]. In Ghana in 1997, 1087 of the 1247 (87.2%) general physicians worked in the urban regions, although 66% of the population lives in the rural areas [9,10]. At a recent OECD meeting of experts on human resources planning, the 20 countries represented reported maldistribution problems [11]. But unlike poor countries, richer ones can mitigate the effects of maldistribution through strategies such as transfer by air or telemedicine.

The imbalanced distribution of health personnel can contribute to great disparities in health outcomes between the rural and urban population. In Mexico, life expectancy for the rural population is 55 years, while in urban areas it is 71 years. In the wealthier northern part of the country, infant mortality is 20/1000, compared to 50/1000 in the poorer southern states [12].

Urban areas are more attractive to health care professionals for their comparative social, cultural and professional advantages [13]. Large metropolitan centers offer more opportunities for career and educational advancement, better employment prospects for health professionals and their family (i.e. spouse), easier access to private practice (an important factor in countries where public salaries are low) and lifestyle-related services and amenities, and better access to education opportunities for their children [6,14,15]. In addition, the low status often conferred to those working in rural and remote areas further contributes to health professionals' preference for settling in urban areas, where positions are perceived as more prestigious [16,17].

While it is in the most remote and underserved areas that health problems are more prominent, this being particularly true for low-income countries [6], urban, wealthier areas often report having too many staff, particularly doctors, as in Côte d'Ivoire, where some doctors remain unemployed in Abidjan, the principal city [18]. Overstaffing in urban areas can lead to underuse of skilled personnel while increasing the total cost of health care system. Paradoxically, instead of encouraging movement of staff towards rural areas, excess numbers of health professionals in urban areas often promote external "brain drain", as professionals start leaving for employment opportunities abroad.

In the last 15 years, as they engaged in reform initiatives aimed at addressing issues of equity in health care and improving the health status of the poor, policy-makers faced the challenge of ensuring that remote and poorly served areas are staffed. Few countries, with notable exceptions (Brazil, Cuba, Iran, Thailand), however, addressed the issue in a systematic and comprehensive manner, and piecemeal interventions have produced rather disappointing results.

Our objective is to offer a better understanding of the determinants of the geographical distribution of health personnel, and to identify and assess strategies to influence it. We review the recent literature on determinants and barriers, and the effects of strategies that attempted to correct geographical imbalances, with a focus on low- and middle-income countries, but also on lessons from richer countries. An analysis of determinants of success and failure of strategies implemented is included. The research for this article relied on an Internet search of web sites and publications on health professionals worldwide and a review, covering the 1995-2004 period, of documents, publications and unpublished reports on distribution of human resources for health.

### Approaches to understanding geographical imbalances

Two main approaches to the geographical distribution of health personnel have been identified: economic and normative. From the point of view of economics, the distribution of health professionals is a function of the health care labour market. Imbalances arise when there is a disequilibrium between supply and demand for labour in a given geographical area. From this perspective, as real wages increase, more health professionals will be willing to be employed and more people will enter the health professions, leading, in the long run, to a new equilibrium and a more balanced distribution of health professionals. This theory predicts that imbalances of health workers can be prevented by establishing a competitive labor market [11].

However, it has been shown that economics is just one factor affecting a health professional's decision as to where to locate his/her practice [19]. Professional, personal, educational and social/lifestyle-related factors can greatly influence job-related decisions. It has also been shown that the health care labour market is not a competitive market, since there usually are substantial entry regulations, information asymmetries and other market failures [11,20].

The normative view defines imbalances in terms of comparison of a certain staff density with some standard or social norm [11]. It leads to emphasizing the role of planning in achieving a balanced distribution of human resources for health (HRH). The norm of reference can be one defined by professional organizations, by government policy, or simply by using a certain region as a comparator. From that perspective, variations in professional density from the defined standard across a geographical area are considered imbalances. By definition there is subjectivity involved in establishing these standards, as well as methodological problems such as defining what is a doctor – or even more complex, what is a nurse (definitional issues arise from variations in how different jurisdictions define the scope of practice of health professionals).

Normative approaches usually use full-time equivalent (FTE) ratios of doctors, nurses, etc., to population. This also has serious limitations, as it says nothing about the productivity of personnel or about the needs of the population, two variables that can show huge differences between countries and within the same country.

Far from being contradictory, the normative and the economic perspectives complement one another. While the normative view focuses on the need and supply side of the health labour market, the economic view tackles the demand and financial incentives needed so that demand will match supply [21].

Standard location theory has been used to predict and explain choices of practice location by health professionals [11,22,23]. It uses the concept of utility function to describe locational preferences of health professionals [22]. The utility function assumes that a number of factors can affect the relative attractiveness of a certain area and play a role in a professional's decision to locate his/her practice [11], a choice decided on the basis of the alternatives that maximize one's utility.

Income is only one variable at work. Dionne [22] has found that quality of leisure, distance to central cities, average income and presence of a hospital significantly increase the probability of having at least one physician in a given town. A study in Norway found that younger physicians tended to prefer leisure to higher income [24]. The same study found that physicians who reported high workload stated a desire to move to an area where workload was lower, while physicians with fewer patients did not express a desire to move [24]. The implications of the interdependence of factors affecting job-related decisions is that the distribution of health professionals may not follow demand only, but also amenities.

The geographical dispersion of health professionals has also been studied through the analysis of average distance circles that map professionals' changing mobility over time [25]. This model assumes an interplay between individual factors to locate practice and a given structure (e.g. medical education). This model can facilitate understanding of the implications of community-based training on ability to retain personnel, changes in the mobility of male and female providers and career trajectories for different health professions [25].

### Determinants of variations in the geographical distribution of HRH

Variations are the result of a mix of decisions by individuals, communities and governments, which are in turn influenced by personal, professional, organizational, economic, political and cultural factors. Rural-urban inequities, inadequate medical education systems, migration, public-to-private brain drain and inadequate payment incentives are just some of the factors identified as contributing to an imbalanced supply of health personnel.

These factors often interrelate and affect one another in many ways. For example, inadequate remuneration and working conditions result in personnel resisting redeployment, as well as promoting rural-to-urban migration [26]. As health professionals concentrate in urban areas and seek career advancement there, they may soon opt to work in the private sector, which may be the reason to move to an urban area in the first place. Consequently, rural-to-urban brain drain is compounded by public-to-private brain drain [26]. Ultimately, the inequitable socioeconomic development of rural compared to urban areas presents the main constraint for achieving a balanced distribution of HRH [13].

Bilodeau and Leduc [27], when discussing factors affecting retention of health personnel in rural and remote areas, define three categories of factors affecting health personnel's motivation to practise in these locations: personal (age, gender, education, etc.), professional (specialization, working hours, incentives, etc.), and contextual/environmental (community amenities, quality of life, population's educational level, etc). The authors further define three distinct decisional phases affecting the retention of health professionals in rural and remote areas: attraction, installation and maintenance. Attraction is defined as "a positive attitude regarding the exercise of medicine in rural and isolated areas, which does not necessarily conduce to installation." Installation consists in the realization of attraction and the decision to practise in a determined area. Maintenance of practice takes place as a result of experiencing living and working in a given area. In each decisional phase, various personal, professional and contextual/environmental factors shape the individual's experience and consequently the decision to relocate.

In our literature review, we identified at least five categories of determinants that affect geographical distribution: individual; organizational; factors related to the health care and educational systems; institutional structures; and the broader sociocultural environment.

### Individual factors

These include a person's social background, ethnicity, age, gender, education, values, beliefs, etc. Growing up in a rural community has been associated with higher probability to practise in rural areas [19]. Women are less prone to accept rural posts and are underrepresented in rural areas [28]. Younger individuals typically have fewer family responsibilities and are more prepared to move or migrate. The presence of family members in rural and remote areas increases the probability that an individual will consider these areas for the establishment of his/her practice [27]. The decision of where to practice is also influenced by an individual's expectations and career advancement plans.

As more females enter the medical profession, the need to understand gender-related differences in terms of specialty preference, geographical location of practice and other characteristics becomes increasingly important. An increasing female medical workforce may not result in more physicians working in rural areas. Comparisons between male and female physicians in the United States have shown that women tend to prefer urban locations, where they have access to salaried work in institutional settings [29]. A study in Bangladesh found that female doctors rarely live in the same village as their assigned post and have higher overall absentee rates. The study suggests that married women doctors will likely live where their husband's jobs are [30].

With women being less likely to accept positions in remote areas, the changing gender composition of health professions has the potential to affect the supply of personnel to rural areas and alter the impact of strategies developed to correct imbalances. In addition, this gender differential has important policy implications, as in many places in the world women are not allowed to be seen by male doctors, making an already skewed availability of health care services even worse for rural women [30].

### Organizational environment

Management style, incentives and career structures, salary scales, recruitment, posting and retention practices are some of the organizational factors that can influence the geographical distribution of personnel. In poor countries, remuneration is usually low and working conditions unsatisfactory. Remuneration, in particular, seems to constitute the most basic influence on retention of health professionals [31]. Health workers often resort to coping strategies, such as adding private practice to their public employment, to overcome unsatisfactory remuneration and working conditions [32,26]. A study in Bihar, India, found that three of four medical officers assigned to a health post were not present in the month of the researchers' visit, but still drew their salaries. Two doctors did not live near the post location and were reported to be busy with their private practices elsewhere. The officer in charge did not complain because the presence of other doctors would interfere with his own private practice [33]. In Angola in the mid-1990s, doctors could earn the equivalent of their weekly salary in one hour of private work [34].

It has been proposed that the low numbers of physicians in rural area has more to do with retention than with recruitment [35], as heavy workloads and professional isolation act as stimuli to look for better working conditions. In Australia, average weekly hours worked are higher for rural practitioners [15]. In Indonesia [6] and Thailand [36,37], rural development plans successfully placed health centres and hospitals in most districts. However, lack of concomitant efforts to deploy personnel to new facilities and retain them resulted in work overload for doctors in rural districts, further pushing them to urban areas or outside the country.

Lack of equipment and supplies and of appropriate facilities can act as a deterrent for health professionals to accept positions in rural and underserved areas. This was a primary reason cited by medical students for not practising in rural Pakistan [17]. Lack of transparency and of due process in the management of postings and promotions is also an incentive to avoid working in remote areas where one gets forgotten.

### Health care and educational system determinants

#### Education and training processes

How health workers are educated can affect the distribution of health personnel in a given area. Resources invested in education and training, role models and contents of training have been linked to the distribution of health professionals in many ways. The location, structure, recruitment methods and criteria of medical schools, for example, have been shown to influence the choice of specialty and location of practice [38].

The predominantly urban-based, curative and specialized care and hospital-centred model of medical education has a direct impact on the composition of the physician workforce and preferred location of practice. Those who select specialized disciplines opt for urban practices in greater proportion, if only for securing access to the infrastructures they need to conduct their practice and to the pool of potential clients [38,39]. Some countries, such as Canada [40] and Brazil [41], have successfully adopted policies to reverse the trend towards specialization, with positive results in terms of geographical deployment of physicians.

The location of a medical school has also been associated with specialization and choice of location of practice. Graduates from medical schools located outside the major urban areas are more likely to practise in rural areas and to select a primary care specialty, such as family medicine [38].

Health professionals practising in remote areas often complain of the lack of opportunities for continuing education and career development, which is crucial in the context of health sector reforms and changing national needs. The education and training of health professionals is an essential component for the development of human resources [42].

#### Health care system

The characteristics of the stock of trained health personnel, such as its volume (number of individuals), its composition by sex, age and occupation, and the dynamic of its evolution, are critical factors in balancing their geographical distribution. Shortages of health personnel, measured by the number of unfilled positions, exist both in developing and developed countries. In the United States, 126 000 full-time positions for registered nurses remain vacant and the national shortage is expected to increase to 400 000 by 2020 [43,44]. Shortages are also reported in the United Kingdom and Canada [14]. They are greater in rural areas [11], and this initiates a "domino" effect: rich countries recruit foreign workers to fill rural positions and professionals leave rural areas to fill in the gaps in cities in the exporting countries [45].

Countries such as Oman and Saudi Arabia have continuously recruited foreign workers to fill crucial gaps [5], also contributing to shortages in poor countries, such as Bangladesh, which have sent part of their production of doctors and nurses to the Gulf for many years. These policies are now being gradually reversed, because their long-term sustainability can no longer be assured [46]. The problem is of particular concern in Africa, where shortages have amplified in the last years, as in Burundi, Ghana [47], Kenya, Mauritania [14] and Zimbabwe [48].

The ageing of the nursing workforce has serious implications for the future of the nursing labour market [49], especially as it combines with declining enrolments in nursing schools, resulting in fewer young women entering the registered nurse workforce and nurses leaving the health sector, due to dissatisfaction with working conditions [50].

HIV/AIDS can affect HRH by reducing the supply of health providers through death or reduced performance or from professionals' leaving the health sector, and by increasing demand for services, which results in increased workload [51,52].

A diminishing stock increases demand in the cities and then contributes to the rural-urban migration. It triggers a vicious cycle through increasing the workload of those who stay and encouraging them to look at migration as a strategy to improve their lot.

#### HRH policy formulation process

The failure to correct imbalances or at least to prevent their occurrence is frequently blamed on the lack of both political commitment to do so and of a favourable economic environment [53]. Another explanation connected to the latter argument relates to the HRH policy formulation and strategy development process itself. HRH planning has often not received adequate attention, even in countries committed to health sector reform. In many countries, progress has been made in recent years to develop national policies for HRH, but the implementation, monitoring and evaluation of these policies have often been slower and more difficult [53]. In Africa, few countries have comprehensive human resource for health policies and plans. Even where there is one, funding does not always follow, and issues of retention and remuneration remain unaddressed [54].

### Institutional environment

The structure, organization and role of national institutions such as the civil service and ministries (education, finance, etc.) are also shaped by a mix of external and national influences. In turn, they influence what happens in the health sector, including the distribution of the workforce. At a broader level of the policy environment, changes such as administrative and political decentralization or civil service reform shape the context in which health services function, including how its personnel are allocated.

#### Decentralization and civil service reform

In recent years, many countries have moved towards decentralization as part of their health sector reform [see 55 for the example of Ghana]. In principle, the transfer of power, resources and responsibilities from central agencies to local units could substantially improve health service delivery [56]. In practice, decentralization also poses important risks and challenges, as it often must be combined with efforts to reform obsolete and bureaucratic civil service structures. It also requires capacities that are not always available [57].

Political will is also central to the success of any decentralization effort. For instance, in Ceara, a poor state in the northeast of Brazil, after decentralization and market-oriented reforms, the state's government implemented a programme of nurse-supervised auxiliary health worker teams serving 84% of the districts. The programme has been associated with a rapid decline in infant mortality, a rise in immunization rates, identification of bottlenecks limiting the use of medical resources and timely interventions to deal with crises [58].

Negative experiences with decentralization efforts have been more common. In the Philippines, the formally centralized national health system had the ability to allocate and distribute health personnel to and from different parts of the country. Local governments in rural areas face difficulty recruiting local health personnel, who prefer to work in urban areas. In addition, as prior efforts to create incentives for rural practice had increased salary and benefits and improved the status of the rural practitioner, decentralization resulted in tighter budgets and an inability of local governments to recruit health workers at the now-higher salaries [59].

### Sociocultural environment

The broader environment encompasses the set of economic, political, social and historical parameters in which the state, governments, social groups and individuals operate. This level contains a national and an international component. Broader environment determinants affect where health professionals will practise by defining basic and fundamental structures and conditions that can either facilitate or hinder a balanced distribution of professionals.

Community and local resources, conditions and opportunities can either draw or repel health professionals to or from a given area. Access to social, cultural, educational and professional opportunities increase preference to settle in particular areas [13].

Value placed by society and family on a profession can affect an individual's choice of a career. In many countries, nurses, primary care providers and general physicians enjoy lower prestige and are less socially valued than other specialists and those working in hospitals, yet they are those more likely to accept to work in remote areas.

Gender imbalances exist in many sectors of the health workforce, with some occupations dominated mainly by females and others – usually those requiring more advanced qualifications – by men. These reflect imbalances in the society in general. In Sri Lanka, for example, women comprise 80% of the nursing workforce [60]. In Bangladesh, women account for only one-fifth of the health services and are mostly nurses; they tend to be absent from decision-making positions [61]. Sociocultural factors often preclude women from accepting positions in rural, remote areas for extended periods. In addition, in countries that impose rural compulsory service as a requirement for graduation and professional certification, women may not be able to graduate or exercise their professions.

Movements towards modernization and industrialization during the 1950s and 1960s led many poor countries, particularly those with newly acquired political independence, to concentrate investments in urban areas, despite that a large proportion of their population was still rural. This form of development resulted in an "urban bias," which in particular brought a concentration of medical schools and health facilities in urban areas. This over-concentration of resources in major centres attracts health professionals in search of better salaries, working conditions and career opportunities [62,63].

NGOs, charitable organizations and traditional practitioners can be a significant source of health care delivery in many countries. In Bolivia, churches provide important services especially in areas of extreme poverty and marginal urban areas; in some areas, churches are the sole providers. In addition, however, almost every rural or marginal urban area has some kind of traditional practitioner. The Bolivian health system is gradually moving to incorporate these practitioners into their networks [64].

### Emigration

Globalization has led to greater mobility and freer movement of the workforce in general. Even though the mobility of health professionals is still generally constrained by regulations at entry, migratory flows from poor and middle-income countries to richer ones seem to be increasing. Consequences seem to be negative for the health sector, as illustrated by the examples of Ethiopia, Kenya and Sierra Leone [66-68]. A variety of pull and push factors influence the movement of health personnel. Push factors within health care systems are low remuneration, work-associated risks (i.e. TB and HIV/AIDS), heavy workloads and poor infrastructure and working conditions. Push factors outside the health care system are political insecurity, crime, taxation levels and repressive social environments. Pull factors include aggressive recruitment by recipient countries and a search for a better quality of life, educational opportunities and higher pay [39].

Even though the migration of health professionals to richer countries can result in some benefits, it contributes to shortages of health personnel in exporting countries [69] and consequently affects the capacity of rural areas to attract and retain health personnel.

Large numbers of trained health personnel have left African countries in recent years [47,14,67,70]. High demand for doctors and nurses [43] in industrialized countries opened great opportunities for highly trained health personnel to migrate. Countries such as Australia, Canada and the United States have been shifting their immigration legislation – by creating temporary visas and expanding availability of work permits – towards increasing their supply of skilled workers [68]. In Ghana, it is estimated that 298 out of 489 doctors who graduated between 1985 and 1994 are living outside the country [47]. The Ministry of Health in Ghana estimated that between 1996 and 2002 the number of doctors in the country shrank from 1154 to 964 [71]. Besides staff shortages, recent trends in migration result in developing countries' facing the financial losses related to the investment in education and training of these professionals [67]. The loss of highly skilled personnel can further affect future generations of professionals, as many academics are among those who leave the country. One study in Ethiopia found that half of the 135 academics teaching at Addis Ababa University in the early 1970s had left the country by mid-1980s [66].

Some benefits arise from migration as well. Temporary migration can help to increase the level and quality of care provided in exporting countries, as returning migrants can bring updated scientific knowledge acquired in international institutions and share new knowledge, technology and techniques with their national colleagues [70]. Workers' remittances can be a significant source of revenue for exporting countries. Remittances account for 24% of Nicaragua's GDP, while in Turkey, remittances are four times larger than the country's inflow of foreign direct investment [72]. Migration can improve the geographical distribution of the health workforce in receiving countries and to a lesser extent in poor countries that receive emigrants. Thirty-one percent of the United Kingdom's health care workforce is from overseas, and 25% of Canadian hospital-based physicians are foreign [39]. In the United States, foreign doctors tend to practise in rural and underserved areas of the country [59]. Cuban doctors have successfully established practice in rural areas of South Africa [36].

### Measures to address geographical imbalances

Many strategies have been tried to prevent or to reduce the maldistribution of HRH. Most have focused on reforming the medical education system and on creating incentives to attract health professionals to otherwise unattractive locations. Financial incentives alone usually have not been sufficient to ensure that remote and underserved areas are and remain adequately staffed.

### Educational reforms

Strategies at this level range from subsidies, review of structure and content of curriculum, the adoption of new pedagogical methods and changes in admission criteria. Decentralization of the location of training institutions has also been proposed, but more rarely implemented. In many countries, medical schools are reforming their curriculum with a view to producing graduates better prepared and more willing to work in underserved areas.

Most reforms emphasize the production of community/family doctors, shifting training towards a primary health care approach. Since the adoption of the "Health for all by the year 2000" goal in 1978, WHO has encouraged a shift of emphasis from hospital-based curative care towards community-oriented preventive and curative care [73].

Educational subsidies to individuals are commonly proposed as a tool to augment the number of students where recruitment is difficult [14]. In the United States, a newly signed bill expands loan-repayment programmes for nurses [74]. However, training more individuals may not be the right answer for improving the distribution of health professionals. Trained individuals may migrate, leave their original profession to work in another area, or withdraw from the labour market, which is especially true for women [14]. In Nepal, the opening of new medical schools created an oversupply of doctors in the country. It was believed that overproduction would lead to professionals' moving to rural areas. Even though the programme achieved some success, in general it led to increased emigration of graduate students [75]. This suggests that retention strategies should be combined with increased production.

Another strategy has been the establishment of rural field residencies or internships as a requirement in medical training. In Ghana, rural experience lengthened rural practice [36]. Data from one medical school in Thailand showed that two-thirds of the graduates continued their rural placements after compulsory training there [37]. Control of access to specialist training has also been tried. In Thailand, where students who intend to specialize must complete residency in rural areas, at least one year of practice is required for some specific specialties (i.e. pathology, psychiatry). Rural doctors have special quotas for access to specialization, under the condition that they return to their district positions. Restriction of specialist training has faced strong resistance from medical schools and professional associations [36,37].

Redirecting postgraduate students' training to other areas of specialization that focus on practical skills geared at community level practice with a shorter training period has been implemented in Ghana. The country's ophthalmic nurse training programme effectively improved geographical access to eye care [55].

A strong case has been made for investments in in-service training and continuing education to stimulate the retention of HRH in targeted areas. These programmes should aim to integrate formal education, subsequent continuing education and actual service provision, therefore ensuring that training has strong practical foundations while continually exposing service providers to the latest knowledge and technology [5].

### Use of community health workers and new cadres of health workers

As countries shift towards stronger primary health care systems, innovative approaches that rely on minimally trained health workers have gained increasing relevance. Today, community health workers (CHW) are an integral part of many national systems. Programmes involving CHW have been established in many different settings, from dispersed rural areas in Africa to inner cities in North America [76]. Community health workers are called by many different names, such as health auxiliaries, barefoot doctors, village health workers and health agents, among others. Their primary role is often to perform preventive medical services, provide basic curative services and to serve as a link between the community and the national health services.

In Bangladesh, starting in 1984, community health workers were incorporated in a tuberculosis control programme initiated by the Bangladesh Rural Advancement Committee (BRAC) to cover underserved areas [77]. CHWs, mostly illiterate women, covered about 200 households under the supervision of paramedics. CHWs directed suspected patients for sputum tests and followed up on their treatment. When compared to the government-run TB programme, the BRAC initiative achieved the same satisfactory cure rates at 50% less cost.

The preventive potential offered by CHWs can affect the pattern of use of health services, reducing the number of medical consultations and hospitalizations [78]. Another project using CHWs in southeastern Brazil was found to have considerably reduced the occurrence of morbidity and mortality among children under five years of age. When compared to similar areas that did not use CHWs, utilization of health services for children under five was 77% higher with CHWs [78]. However, the mere inclusion of CHWs may not be a guarantee of the success of an intervention. In Burkina Faso, the use of CHWs was found to remain low. Household surveys indicated that severity of the disease and perceived effectiveness of the treatment were the most important determinants of health-seeking behaviour [79].

The effectiveness of CHWs has been the focus of many discussions. In general, well-designed programmes using well-trained CHWs, with proper support and supervision and with a clear, defined role for CHWs have been successful [76-79]. CHWs projects need to take into account the culture, geography and socioeconomic context of the communities to which they are directed.

As certain types of health professionals are more resistant to accepting positions in rural and deprived areas, some argue that new cadres of health workers should be developed to take over some of the responsibilities normally held by doctors or nurses [5]. It is expected that multipurpose health workers such as medical assistants will be more willing to work in rural and remote areas, but this must be verified. This strategy is often resisted by doctors, who do not agree that others than themselves should be allowed to perform certain procedures, such as removal of retained placenta, caesarian sections, even drawing blood [59]. This monopoly of tasks contributes to limiting the availability of health services areas with high population per doctor, which is dramatic when it concerns emergency services.

But there are some exceptions, such as in Malawi and in Tanzania, where paramedical staff who are able to provide urgent surgical interventions have been trained [31]. In Argentina, lay nurses are receiving training to work as nursing auxiliaries [64]. In Ghana, a new curriculum has been developed for existing cadres. Community health workers are now trained to have midwifery skills and the "Life Saving Skills" project trained rural midwives with skills normally reserved to physicians [55]. Ghana has also created shorter specialist training programmes [39]. Ghana's Health Strategy for 2002-2006 includes periodic reviews of professional training courses with a view to making them relevant to country needs [71].

Even though staff retention may not be addressed by delegating skills to other health professionals, some greatly needed services can be ensured and sustained by these measures [31]. In Botswana, training more nurse practitioners and pharmacists has compensated for the lack of physicians in some areas [53]. In Ethiopia and Mozambique, "field surgeons" and "clinical officers" provide a substantial amount of health services normally expected to be handled by doctors [31].

Laos Red Cross in Attapeu, under a grant from British NGO Health Unlimited, and CIDA (Canadian International Development Agency) provided training for 100 village health workers, most of them from poor ethnic minorities. These village workers can now respond to health emergencies and provide basic first aid to remote communities in mountainous parts of Laos, where health services are precarious [89].

Iran almost eliminated the differences between rural and urban rates of infant mortality between the mid-1970s (120 per 1000 in rural areas and 62 per 1000 in urban areas) and the mid-1990s (30 per 1000 in rural areas and 28 per 1000 in urban areas), by establishing a network of "rural health houses" (khane bedash), staffed by workers (behvarz) recruited from the community, trained to offer basic child services, to record health information and to refer more complex cases to rural health centres covering five health houses. This was done at low cost, using simple but well-managed tools, such as an integrated health information system. Spectacular reductions of maternal mortality were also achieved: from 120 to 24 deaths per 100 000 live births in urban areas, 370 to 55 in rural areas [90].

Even though the role of auxiliaries and community health workers in increasing coverage has been recognized, the use of alternative health professionals has met strong resistance from some professional groups. In Ghana, the Village Health Worker system and the idea of training traditional birth assistants has been questioned and opposed by professional associations such as the National Registered Midwives Association. In response, the country is moving towards reducing and reprofiling existing cadres into multipurpose health workers.

### Rural recruitment and training

Rural recruitment has been promoted in some medical schools with relative success. In Thailand, the government has aimed to annually produce 300 doctors specifically for rural areas. Students are recruited through mechanisms that require them to sign contracts for residency with their provincial health office. Their contract mandates two to four years of public sector employment after graduation. A network of local health clinics and hospitals where students can train has been established to support the programme. Schools are distributed throughout the country and students receive a highly subsidized education. Students conduct their practical training at the location where they will work after graduation, to familiarize themselves with their future working environment. The programme considerably increased the proportion of students of rural origins [36,37].

Rural recruitment, however, also presents at least two problems. Rural students from poor families often have more difficulties in passing competitive examinations and in keeping up with the demands of medical education, and most rural students tend to come from the better-off local families [36,37].

Another example is from Cuba, where the "Escuela Lationoamerina de Ciencias Medicas" focuses on recruiting students from low-income families, indigenous communities and underserved areas in the Americas and Africa [59]. The effect of this strategy has not been measured, however.

To address the issues of geographical and specialty maldistribution of physicians in the United States, Jefferson Medical College implemented the Physician Shortage Area Program (PSAP) which combines a selective medical school admissions policy and a special educational programme [80]. The PSAP focuses on selecting medical school applicants with rural backgrounds who intend to practice family medicine in rural and underserved areas. Students admitted to the PSAP programme are awarded more financial aid than other Jefferson students. The programme includes a family medicine faculty advisor, a required third-year clerkship in family medicine in one of two non-urban areas and an internship in family medicine.

Evaluations conducted in 1993 and 1999 [80,81] concluded that the PSAP successfully increased the number of family physicians practising in rural and underserved areas of Pennsylvania. The 1999 evaluation [81] also demonstrated that the effects persisted over time. Of PSAP students who graduated 5 to 10 years before the 1999 evaluation, 87% remained practicing rural family medicine and 94% still practised in underserved areas.

Centrally located medical schools draw students and health care services to urban centres. Thus, the establishment of regional medical schools is expected to promote a more equitable distribution of health professionals and services. The Thai experience suggests that regional medical schools and a focus on recruiting rural students can improve the distribution of health personnel in rural areas [37].

### Integration of training, education and service

Calls for a broader responsibility of health education institutions in improving community health and building linkages with other community stakeholders have been made [73]. New approaches include community-based education, integration of primary health care and public health, multiprofessional education and problem-based learning. A group of representatives of health care educational institutions founded The Network: Towards Unity for Health (TUFH), which has become "a global association of institutions for educating health professionals to be committed to contribute, through innovative education, research, and service, to the improvement and maintenance of health in the communities they serve." [73]. The Network has 160 members from more than 80 countries.

In Latin America, for example, the UNI programme (A New Initiative in the Education of Health Professionals – Links with the Community) promotes cooperation between universities, health services and communities. The goal of the programme is to promote concomitant changes in universities, health services and communities, as well as in their relationships [82]. This process involves four objectives: (1) changes in the process of education through curricular reform emphasizing interdisciplinary approaches and real-life experiences; (2) changes in health services to become effective, integrated and oriented towards the real needs of the population served; (3) strengthening citizenship and popular participation in order to increase the community's access to knowledge and technology; and (4) establishing more democratic, flexible and relevant relationships between institutions involved in the provision of health care [82].

Launched in 1993 in Brazil, the UNI project is now on its second phase, with 23 projects implemented in 11 Latin American countries [83]. The UNI programme has been recognized for promoting significant changes in how health services are offered and used in the communities where it has been implemented, including increased user satisfaction, increased community participation in decision-making processes, improved collection and use of information and more integration between hospitals and primary health care centres [84].

### New educational tools

The globalization of communications and advances in informational technology have greatly increased the scope of educational possibilities. Virtual universities, networks of institutions and professional associations, international standards of certification and distance learning are now possible avenues for improving a country's capacity to educate and train its health workforce [65], even when they practice in isolated areas.

New technologies such as telehealth and telemedicine have the potential to increase the supply of health professionals to rural and deprived areas by facilitating professional collaboration and development, by supporting, for example, continuing education and access to some services (interpretation of x-rays, specialist opinions). The use of videoconference technology has opened many prospects for those working in rural and remote areas, as a treatment and diagnostic tool as well as a means to gain access to education and training over long distances.

In Australia, a videoconference training initiative developed for professionals working with at-risk youth in rural and remote areas reported high levels of satisfaction among participants and a decreased feeling of professional isolation [85]. Taiwan is currently developing the Cyber Medical Center international collaboration project, which aims to create a network system that will allow teleconsultations and online continuing education [86]. In Thailand, the launch of the country's first communications satellite allowed for the implementation of a nationwide telemedicine network by the Ministry of Public Health, currently linking 19 hospitals with health facilities all over the country [87]. The relative cost and benefits of these new technologies are yet to be assessed, as well as issues related to the protection of privacy, service standards, licensure and liability insurance coverage.

### Regulatory and administrative measures

Contracts that require a certain number of years in public service, especially when training is state-sponsored, have been implemented in many Latin America countries and in the Philippines, Tanzania and Thailand. Students must spend a variable number of years in a designated area or pay fines [36]. Overall, geographical distribution has been little affected by such policies [5].

In Mexico and Thailand, failure to adjust to high inflation rates resulted in fines that can be easily paid. Increasing the fine or imposing fees that must be reimbursed has met strong resistance from medical schools and professional associations.

Some countries implemented high tuition fees for medical students. Those who cannot afford to pay are awarded educational loans from the government that can be paid back after graduation either in cash or by public service work. In Indonesia, South Africa and Viet Nam students receive their diplomas only after completing their public service period, which may entail practising in rural areas.

Many Latin American countries have implemented a system of compulsory rural internships for new graduates. Initially designed for physicians, the system was later extended to nurses, social workers and other health professions. Evaluation of these programmes pointed to the low acceptability of the systems by the students, which resulted in low productivity [59].

The prevention of emigration by establishing bonding measures has been tried in a number of countries in the hope of increasing the number of health professionals in rural and remote areas. In general, the system has been considered unfair. Since other careers do not require compulsory service, bonding may lead individuals away from medical education. In addition, it can be particularly problematic for women, who are often unable to accept remote positions [88]. In countries where women comprise a considerable part of the health workforce, bonding systems can result in a significant portion of needed personnel not being able to complete their graduation requirements or engage in professional practice [36].

The effectiveness of strategies of compulsory "social service" after graduation in less attractive regions must be assessed. If the process of posting is not transparent, it opens the door to corruption and becomes ineffective. If it is rigorous, it can turn out to be an incentive to emigrate from the country.

### Financial and professional incentives

Multiple incentives to make working in unattractive areas more appealing have been proposed with variable success. More generous benefits, such as health insurance and vacation time, are the most commonly used incentives. Other benefits may include tuition reimbursement, flexible work hours, bonuses based on experience or length of commitment, study and recreation leaves, employment opportunities for doctor's spouses, better accommodation facilities and improvements in educational institutions for doctor's children [14,39].

In Thailand, financial incentives started with special allowances for physicians working in remote district hospitals in 1983. Monthly allowances were USD 88 for those working in regular districts and USD 108 for those working in remote districts. The latter were restricted from accepting travel per diem or on-call payments. These restrictions were lifted in 1994, and a non-private practice allowance was established in 1995. Physicians who agreed not to engage in private practice received an extra USD 400.00 per month [37]. However, these incentives still added up to less than physicians could earn in private practice.

In the Philippines, the Magna Carta for Public Health Workers was created with the intention of making rural positions more attractive. The package included increased salaries and benefits, particularly for physicians. The programme was further strengthened in 1993 with the launch of the "Doctors of the Barrios" programme, which doubled the benefits for doctors willing to relocate to remote areas. With the 1993 devolution of health services, most local governments found themselves unable to hire at the prevailing high salary levels. As a result, professionals started moving back to urban areas and to apply for work through the national agency instead of local government offices [59].

Possibilities for doctors to work privately in public institutions are being offered in some countries to neutralize an ongoing drain of qualified staff from the public sector. This strategy is considered successful in Bahrain, but the experiences of Ghana and Nepal show that such incentive can lead to the diversion of scarce resources from public services and can induce professionals to end up opting for independent private practice [14]. In Indonesia, private practice is allowed as a means to make up for low salaries. The practice results in health professionals' selecting positions in locations where the potential for private income is higher, usually in urban, higher-income areas [88].

In Quebec, Canada, the government agreed with unions representing doctors on a mix of incentives to improve the regional distribution of the medical workforce. Fees are raised by 15% to 25% in regions considered as underserved and reduced by 30% in regions considered as having an oversupply. Some other benefits are offered to those accepting to work in remote areas, such as subsidies to settle a practice and access to continuing education [40]. This has been shown to improve the distribution of general practitioners, but has had less impact on the choice of location of practice of specialists, particularly those whose practice requires access to technology available only in specialized hospitals. It appears that economic incentives are not enough to influence redeployment; they must be supplemented by other incentives, mainly professional [27].

In Indonesia, graduates who work in the very remote areas receive a higher salary and the guarantee of a civil service career after the completion of the three-year compulsory contract [6]. A civil service career is highly desirable, since it allows for private practice in the evenings as well as free access to specialist training [88]. This practice, however, has been criticized for attracting the wrong types of health professional into rural areas. Those who are interested in specialist training often have no interest in public health work in remote areas and leave soon after the completion of the compulsory contract. In addition, since they are required to complete their rural service before specialization, doctors are able to complete their training only in their late thirties or early forties. Therefore, the practice significantly reduces the returns of the investment placed in their training.

In many countries, strategies to deal with inequitable distribution of HRH lie beyond the scope of the ministry of health. Major civil service reforms are necessary in order to better distribute health professionals in the public sector. In Indonesia, for example, the centralized budget allows staff to migrate to preferred locations in developed areas, without adequate control, planning or supervision at the provincial and district level [88].

### Better national policies and international agreements

The government of Quebec, Canada, implemented a series of policies to control the growth and modify the structure of the medical workforce, by controlling admissions to medical school and defining quotas for specializations based on predetermined regional needs [27]. In the early 1990s Brazil developed a strategy, which now has been adopted by more than 80% of municipalities, to give access to basic services in poor and remote regions – some 63 million as of mid-2004. "Family health teams," composed of a medical doctor, a nurse, an auxiliary and four to six community health workers "and aiming at dealing with 85% of health problems in the municipality, have been trained and deployed all over the territory, thanks to incentives attractive enough to convince health workers to join in. Thailand has engaged consistently and in a flexible manner in HRH policy for 40 years [37], which may explain its relative success in dealing with deployment issues.

Strategies to improve social and professional recognition for health professionals in remote areas have been devised and implemented [88]. They aim at improving the morale of rural doctors, encouraging them to stay in rural areas. In Thailand, rural doctors created their own society, "The Rural Doctor Society," which provides innovative programmes to support rural doctors. The society was very well accepted by the public and the medical profession. In addition, public recognition awards were established, including an annual hardship award given to the best doctor in the most remote location, and the "best rural doctor of the year" award. Some doctors received honorary master's and Ph.D degrees from universities. At the national level, rural doctors have been recognized as "the model Thai of the year." [36,37].

### Factors affecting success/failures of strategies dealing with geographical distribution of HRH

Key determinants of successful strategies include: length of time on the national priority agenda, long-term political commitment, integration of efforts with those of other sectors such as education and civil service and ability to reconcile different expectations from varied stakeholders. The importance of involving the key actors in the policy formulation and implementation process stands out as a crucial element in the success of a policy [53,91]. Emphasis must be placed on bringing together different stakeholders at the stage of developing policy options.

Factors associated with negative outcomes are lack of resources, lack of understanding of cultural context and resistance from professional or social groups. Decentralization and economic crisis can create conflicts between health organizations, political forces, unions and professional associations. Professional associations and workers unions can feel threatened by changes that affect long-established privileges. These groups can be powerful enough to delay or reverse changes [59]. The need for a stable social and political environment cannot be overemphasized. For example, in Ghana in 1977, 1984 and 1995 no graduates were produced due to closure of university as a result of political unrest and lecturers' strikes [55]. This also happened in Mali in 1993-94 and in Senegal in 1986 and 1992.

Systems of incentives are often central strategies employed by governments to correct imbalances [92]. However, the degree of success of such incentives can depend largely on factors not directly related to the health sector. Poorly targeted financial incentives can also have undesirable effects, as shown by experiences in Mexico and Thailand. In both countries, financial incentives for rural work resulted in an early departure of professionals from rural areas, by making it possible for them to pay the fine to break compulsory service. In some cases, complementary measures may add effectiveness to incentives. Bahrain, Ghana and Nepal allowed after-hours private practices as a way to contain public-to-private brain drain. The measure raised many concerns about the quality of care and effort dispensed in the public system, however. In response, the three countries implemented sets of standards and controls to prevent this negative effect from taking place [92].

### Policy messages

The geographical distribution of health personnel refers to their spatial allocation. It is said to be imbalanced when a norm is applied, such as population/personnel ratios, or more sophisticated needs-related indicators. Geographical distribution matters a lot, since it determines which services, and in what quantity and quality, will be available. Imbalances raise problems of equity (services not being available according to needs), of efficiency (surpluses/shortages) and of effectiveness of services, let alone of satisfaction of users. The health-related MDGs cannot be achieved if vulnerable populations do not have access to skilled personnel and to other necessary inputs. A perfect balance is probably utopian, but it is conceivable to achieve a better distribution through strategies based on a good understanding of its dynamics.

Workforce issues are likely to become increasingly crucial and acute as health sector reforms focus on decentralization and on new public-private partnerships, and as the commitment to achieving the MDGs brings more funds to health sector, like the debt alleviation process. Coherent and well-formulated HRH policies and strategies, as well as the ability to implement and monitor them, are therefore crucially needed [57,91].

At the policy level, one major conclusion seems to be that the geographical distribution of the health workforce cannot be dealt with in isolation. From the country examples included here, it is clear that strategies are often reactive measures in response to a crisis. They are often fragmented, uncoordinated and sometimes inconsistent. They do not always take into account factors residing outside the domain of the Ministry of Health. Strategies must be multifaceted, integrated and coordinated in relation to the health sector and its environment. One major factor is the emergence of the private sector, both for-profit and not-for-profit, which imposes great changes on the environment in which HRH issues are to be addressed.

Highly-skilled professionals and institutions respond more to incentives than to control mechanisms. Systems incentives can be devised after having analysed the expectations of providers, which are likely to be a mix of economic, professional, personal and family-related. In order to implement policies that generate and maintain work motivation, there is a need to better understand its determinants and the potential of certain incentives to produce motivation. Motivation is not influenced solely by specific incentive schemes targeted at workers, but also by the range of health sector reforms that affect organizational culture and structures, channels of accountability, community feedback, etc. [93].

A study conducted among rural health workers in Viet Nam identified motivating and discouraging factors for health worker performance, which encompassed both financial and non-financial incentives. Motivating factors included appreciation by managers and the community, income and job stability, while discouraging factors were mostly related to low salaries and difficult working conditions. These studies suggest that policies oriented at promoting worker motivation need to be aligned with individual, organization and reform goals [94].

Financial incentives are usually not sufficient to improve the distribution of health professionals. Strategies that included efforts to increase social acceptance and recognition of rural health professionals have often been more successful. Examples include the creation of social recognition awards and support groups for rural practitioners. Social movements towards acceptance and appreciation of rural health personnel can effectively improve staff morale and retention in rural areas. Such strategies should be encouraged and supported in order to create positive images and motivate young graduates to envisage such postings.

It is important to emphasize that each country's health system and human resources situation is specific and workforce issues are multidimensional and interconnected. As such, contextual factors need to be clearly identified, so that the complexity and interconnection of issues is taken into account when relating to each specific national context. Evaluation studies are thus crucial in order to understand interrelationships between determinants of geographical imbalances and strategies to correct them.

Individual factors have a more immediate influence on the geographical distribution of health personnel, whereas factors linked to the organizational and community environment have an intermediary influence between the broader environmental factors and individual decisions. Actions to influence those factors to ensure that they produce the expected effects will be more complex and difficult, and will require more time, as we move from individual to social factors.

It is easier to change the recruitment criteria at a medical school to alter the profile of future doctors, than to change the incentive system – which might be under the control of an agency outside the health sector – or to change social attitudes towards women. Too often, strategies to address imbalances focus on individual and organizational determinants, which seem easier to influence. These strategies tend to ignore that organizations and individuals operate within a broader context, which can enhance or reduce the ability of governments to implement corrective interventions.

For example, low salaries and unsatisfactory working conditions are often cited as reasons for not practising in rural and remote areas. These are frequently considered to result from cumbersome and bureaucratic organizational and civil service structures. These are, in turn, deeply rooted in larger social, political and economic deficiencies such as political instability, dominance by small ruling classes, a culture of clientelism, poor institutional capacity, etc. To say that improved salaries and working conditions are the strategies to attract and retain personnel to remote areas is valid, but they are far from simple to implement, since the problems of low salaries and bad working conditions have their roots in complex organizational and social problems that must be attacked simultaneously and in other sectors.

Another example would be gender imbalances, which influence the geographical distribution because women tend to avoid rural and remote areas. These reflect socially and culturally defined roles for males and females [95] and will be corrected if and when these definitions change. In other words, interventions that attempt to alter basic economic, political and social structures are likely to achieve long-term, sustainable results, but they are much more complex and take longer to produce results. Ultimately, only equitable socioeconomic conditions for rural compared to urban areas, adequate investment in human resources, and stable and legitimate political institutions are the basis for achieving a balanced distribution of the health workforce.

## Competing interests

The author(s) declare that they have no competing interests.

## Authors' contributions

MCF searched the literature. The authors participated equally in the analysis of materials and in the writing of the article.
